# Perceived Environmental Threats and Pro-Environmental Behaviors: Investigating the Role of Political Participation Using a South Korean Survey

**DOI:** 10.3390/ijerph17093244

**Published:** 2020-05-06

**Authors:** Jae Young Lim, Kuk-Kyoung Moon

**Affiliations:** 1 Community Wellbeing Research Center, Seoul National University, Seoul 08826, Korea; jaeyounglim@yahoo.com; 2 Department of Public Administration, Inha University, Incheon 22212, Korea

**Keywords:** perceived environmental threats, political participation, pro-environmental behaviors

## Abstract

Climate change and environmental pollution are increasingly ravaging countries around the world. This study examines the direct effects of perceived environmental threats and political participation, as well as their joint effects, on individuals’ support for a lower standard of living and the increased government spending necessary for environmental protection. Using the 2014 South Korean General Social Survey and an ordered probit, the study finds that individuals’ perceptions of environmental threats are associated positively with their support for government spending and a lower standard of living. Political participation is statistically significant and positive only in its relationship with support for a lower standard of living. Nevertheless, political participation is a powerful moderator and amplifies positive relationships between individuals’ perceptions of environmental threats and their support for a lower standard of living and government spending on environmental protection. In estimating predicted probabilities of strong support, perceived environmental threats and political participation jointly increased support for lower living standards by 35.67% and for government spending by 69.58%.

## 1. Introduction

The Earth’s average temperature is rising. In recent years, climate change and pollution have been wreaking havoc in countries around the world [1,2,3]. The increasing number of storms and fires are testing countries’ disaster preparedness and response, demanding untold labor and sacrifice from the citizenry. Pollution threatens the sustainability of human life, and the sight of plastic debris filling the oceans no longer seems newsworthy [4]. In the words of a popular climate change author, “It is worse, much worse, than you think” [3]. The consequences of global warming may be inevitable, but efforts are being made to mitigate and adapt to them. Pro-environmental behaviors can be considered the manifestations of mitigation efforts, whereas adaptation requires changes in political and social systems in reaction to the consequences of global warming and pollution [3,5]. This study focuses on two types of pro-environmental behaviors among individuals: supporting a lower standard of living and advocating the government spending necessary for environmental protection.

The emphasis on lifestyle changes in reaction to environmental degradation reflects citizens’ role as customers in modern society [6]. Citizens’ lifestyles are increasingly viewed in terms of the citizen-customer framework [6,7]. Spaargaren and Mol view the framework as the “post-modern alignment of two fundamentally modernist concepts” [7]. They argue that citizenship, which was previously acted upon and represented through the government, has morphed into consumer citizenship [7]. The citizen-customer discourse has also flourished thanks to conservative policies and the deep penetration of privatization into social welfare policies in recent decades [6]. In developing a positive environmental outlook, policymakers are also increasingly relying on citizens’ individualistic changes [6]. Several studies have considered public policies through the lens of consumption choices, examining consumption options for reducing greenhouse gas emissions [8], customers’ energy lifestyles and consumption [9], tourists’ consumption behaviors [10], lifestyle-related strategies to mitigate climate change [11], and the link between lifestyle and climate change [12]. Similarly, support for the level of government spending necessary for environmental protection can be viewed in terms of the citizen-customer framework. Citizens can choose to direct their support to other matters, such as the economy and infrastructure; in doing so, they show their consumption preferences for certain items. Thus, when government coffers are finite and increased spending in one area can crowd out spending in other areas, support for government spending for environmental protection is a choice that citizens, as customers, make to mitigate climate change and pollution.

What, then, may motivate individuals to exhibit pro-environmental behaviors, such as supporting a lower standard of living and government spending for environmental protection? Among many predictors of such behaviors, the study narrows its focus to perceived environmental threats and political participation. A sizable number of studies have explored diverse ways to identify the determinants of pro-environmental behaviors. Oriented in environmental behavior and education, they have identified several crucial factors, including knowledge, values, attitudes, feelings, perceptions, demography, and external factors [13,14,15,16,17,18,19,20,21,22]. While these studies have emphasized the role each factor plays in influencing pro-environmental behaviors, few have examined the joint effect of threat perceptions and political participation as a proxy of self-efficacy and locus of control, as investigated in this study. Thus, the study fills a crucial gap by addressing the joint effects of multiple factors.

Defined as the likelihood of adverse consequences of an environmental event [23], perceived threats can influence individuals’ behaviors and behavioral intentions [23,24,25]. A high degree of perceived environmental threat can heighten the sense of threat severity and vulnerability among individuals, resulting in protective actions by individuals to ameliorate such threats [26,27].

Political participation lies at the heart of citizen-led participatory democracy. It imparts upon individuals a sense of political efficacy, the feeling that individuals can bring about political change [28]. Individuals with strong political efficacy are more likely to possess internal efficacy to effect personal changes as well as external efficacy to demand proactive responses from government [29,30]. Political participation is even more crucial when it comes to taking the pro-environmental actions needed to help address the fundamental challenges posed by the current environmental crises. Thus, individuals with a higher level of political participation are more likely to engage in personal pro-environmental actions and espouse the need for government spending to protect the environment.

Political participation also serves as a powerful moderator for those who perceive a high level of environmental threat and seek to take active ameliorative actions. Politically active individuals likely have a high degree of internal and external political efficacy to reshape personal choices and political outcomes [29,30]. These individuals will adopt a proactive coping approach to significant environmental threats and are more likely to take personal action by supporting a lower standard of living and government spending for environmental protection.

This study assesses the joint effects of a combination of factors—environmental threat perception and political participation—on pro-environmental behaviors. The study also addresses the value-action gap highlighted by scholars [31,32]. Individuals’ values are not always rational, but they are contested and negotiated [31,32]. Political participation can address the gap by removing barriers to action and linking action to perceptions. With this approach, the study helps enrich an already formidable body of research on environmental behavior.

Using the 2014 South Korean General Social Survey and an ordered probit, the study contributes to the growing body of research on environmental behavior. The paper is organized as follows. First, the paper explores theoretical mechanisms that may explain why perceived environmental threats can increase individuals’ support for a lower standard of living and raised government spending on the environment. Second, the paper examines why political participation produces a similar outcome and the underlying mechanism for this effect, which can moderate and amplify relationships between perceived environmental threats and support for lower living standards on the one hand, and between perceived environmental threats and government spending on the environment on the other. Third, the empirical results are presented along with a discussion of the results and the implications for public officials.

## 2. Perceived Environmental Threats and Pro-Environmental Behaviors

Countries around the world are no longer immune from the ravages of global warming. Increased wildfires in Australia and the United States attest to the severity of climate change [33,34]. Pollution is also a serious concern for some countries. Ultrafine dust and poor air quality are rendering human life less sustainable in some parts of the world [2,3]. Such environmental degradation presages more ominous consequences in the years ahead [2,3], and pro-environmental actions are more urgently needed than ever before. Thus, this study examines the factors that lead individuals to support behaviors such as lower living standards and increased government spending for environmental protection.

Among the many factors that are associated with pro-environmental behaviors, this study focuses on individuals’ perceptions of environmental threats. Environmental threat perception can be defined as the perceived likelihood of adverse consequences produced by an environmental event [23]. This study is built on the premise that individuals’ perceptions of increased environmental threat are associated with increased proactivity among individuals to take ameliorative action to address these threats [6].

The mechanism that links perceived threats to ameliorative action can be found in protective motivation theory (PMT). PMT emerged in studies that sought to understand how threats appeal to individuals and influence their behavioral choices [35,36]. The theory is derived from expectancy-value theory, which contends that individuals’ achievements are driven by their expectations for success and the subjective value they place upon the tasks needed for such success [37]. PMT offers a similar analogy; it is centered on individuals’ expectations of threats and the behaviors they adopt to cope with these threats. In PMT, perceived threats result in two cognitive processes taking place within each individual: threat appraisal and coping appraisal [31,38]. Threat appraisal consists of two constructs, threat vulnerability and threat severity [35,36]. When individuals possess a high perceived likelihood of exposure to threats (threat vulnerability) and a corresponding perception that the consequences of such exposure are likely to be serious (threat severity), they are likely to exhibit stronger protective behaviors, such as pro-environmental behaviors [26,27]. Similarly, threats enable individuals to perform two coping mechanisms: response efficacy and self-efficacy. Protective behaviors are also affected by the ways in which individuals perceive the effectiveness of their behaviors to prevent threats (response efficacy) and the ways in which they rate their capacity to perform such behaviors (self-efficacy) [38,39]. These two cognitive processes—threat appraisal and coping appraisal—form how perceived threats appeal to individuals and impact their behaviors [38,39]. When threats are appraised as substantial, individuals are likely motivated to take protective action [26,27]. This study does not directly investigate coping appraisal, but it is likely that individuals with greater coping appraisal would also be inclined to engage in pro-environmental behaviors to prevent and mitigate threats [26,27].

Several studies have demonstrated positive associations between perceived environmental threats and ameliorative actions. For instance, humans are more likely to take pro-environmental measures when experiencing significant environmental threats and heightened mortality salience, or sense of fear based on the proximity of death [40,41]. Baldassare and Katz showed that residents sensing acute air and water pollution in Orange County (California, USA) took proactive approaches, such as purchasing green products and saving water [24]. Fisher et al. found that residents whose homes were found to contain radon were more likely to take corrective actions than those whose homes did not contain radon [42]. Positive relationships between perceived threats and ameliorative actions have been found in relation to climate change [23] and organic farming [25]. Séguin et al. showed that perceptions of environmental health risks are positively associated with environmental activism among individuals [43]. The fact that perceived threats relate positively to pro-environmental actions suggests a strong possibility of support for the following hypotheses:

**Hypothesis** **1a.** *Perceived environmental threats are positively associated with support for a lower standard of living to protect the environment.*

**Hypothesis** **1b.** *Perceived environmental threats are positively associated with support for the government spending necessary for environmental protection.*

## 3. Political Participation and Its Moderation of the Relationship between Perceived Environmental Threats and Pro-Environmental Behaviors

Political participation plays a vital role in sustaining and stimulating democratic governance. Defined as voluntary individual activities to influence political choices [44], political participation takes on even more urgency in a participatory democracy, an increasingly common governance norm around the world. Political participation requires that citizens partake in active political life; in doing so, they learn how to be participative, and they facilitate democratization [45]. Citizens’ political participation not only strengthens their character development and self-actualization but also has salutary effects on the development of political institutions [45,46,47,48].

What makes individuals’ self-actualization possible is a mechanism called efficacy. Political participation boosts political efficacy, which helps to reshape individual and political outcomes. Defined as the feeling among individuals that they can bring about political or social change [28], political efficacy imbues individuals with a sense of self-control (or internal locus of control) and self-competence [49]. In this way, political efficacy nurtured through political participation facilitates individuals’ efforts to cope with diverse social phenomena. Political efficacy is comprised of internal and external components [29,50]. Internal efficacy touches on individuals’ self-competence in understanding politics and effecting change, whereas external efficacy concerns the way in which individuals perceive the government’s responses to their demands [29,30,46,51]. Bandura indicated that the two concepts are not mutually exclusive [5,6] and discussed how individual efficacy can lead to collective efficacy. When people believe in their autonomy and self-competence, and they share their beliefs with others, these joint efforts pave the way for collective endeavors to solve social problems. The self and the social structure are mutually interdependent, and personal agency is expanded in the social realm, facilitating collective agency for communal actions [49,52]. Thus, political efficacy generated through political participation can individually and collectively motivate individuals to take proactive action to solve communal problems, such as environmental pollution and climate change.

Several studies have demonstrated the positive relationship between political participation and communal action, such as support for environmental measures [19,53,54,55]. History also provides evidence of positive links between political participation and pro-environmental behaviors. Political participation has ushered in environmental protection movements that have helped stop unrestricted pollution and the production of hazardous materials [56]. For instance, widespread use of pesticides led Rachel Carson to publish *Silent Spring* in 1962, which alerted the public to a potential dystopian world with sick humans and no animals [57]. The grim world depicted in the best seller soon led to numerous environmental rallies organized in the 1960s and 1970s, resulting in the U.S. government’s enactment of crucial environmental protection bills, such as the Clean Air Act (1970), the Pesticide Control Act (1972), the Endangered Species Act (1973), and the Safe Drinking Water Act (1974) [56]. Environmental activism was also critical in South Korea, which enacted a series of legislation to address disasters ranging from phenol contamination in drinking water in the early 1990s to toxic humidifier sterilizers in the 2010s [58,59]. Many environmental reforms would not have been possible without people’s political and social activism, which influenced political choices across the political spectrum.

**Figure 1:**
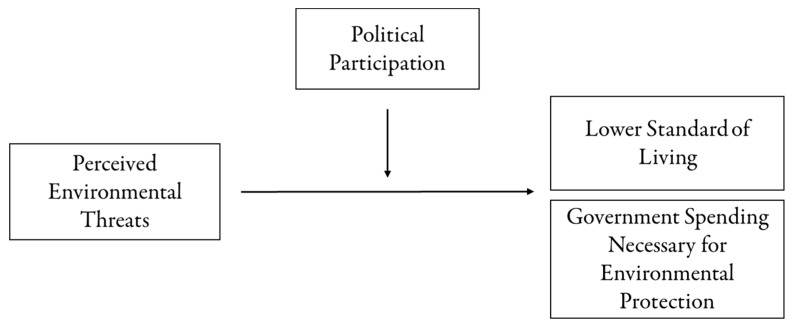
The Conceptual Framework

Additionally, political participation moderates and enhances the impact of perceived environmental threats on support for lower living standards and increased government spending. The moderation mechanism can be explained by the way that political participation can provide coping mechanisms to individuals who perceive environmental threats. As described in Section 2, PMT describes two mechanisms that induce protection behaviors, threat appraisal and coping appraisal [35,36]. Higher perceived threats are likely to motivate individuals to take personal action and support government measures that buttress environmental protection. However, PMT theorists argue that the link between perceived environmental threats and protective actions is likely enhanced when the degree of coping appraisal is high [38,39]. Individuals actively engaged in political participation are more likely to cope with perceived threats positively due to their high political efficacy [49,52,60]. Instead of ignoring or being passive about threats, politically active individuals are more likely to take action personally and demand proactivity from the government [26,27]. Thus, the following hypotheses are posited, with Figure 1 describing the conceptual framework of this study:

**Hypothesis** **2a.** *Political participation is positively associated with support for a lower standard of living to protect the environment.*

**Hypothesis** **2b.** *Political participation is positively associated with support for the level of government spending necessary for environmental protection.*

**Hypothesis** **3a.** *Political participation moderates the relationship between individuals’ perceptions of environmental threats and their support for a lower standard of living such that the relationship becomes stronger as the level of political participation increases.*

**Hypothesis** **3b.** *Political participation moderates the relationship between individuals’ perceptions of environmental threats and their support for the level of government spending necessary for environmental protection such that the relationship becomes stronger as the level of political participation increases.*

## 4. Data and Measurement

The data came from the Korean General Social Survey (KGSS). The survey was first implemented by the Sungkyunkwan University Survey Research Center in 2003 [61]. It closely follows the question format of the U.S. General Social Survey, repeating a core set of questions in every survey and a set of topical questions at regular intervals. The KGSS was implemented every year from 2003 to 2014, but since 2014, the survey has been conducted biennially. The survey relies on a multi-state area probability sampling method to account for population representation and targets South Korean residents ages 18 and older [61]. The present study used the cross-sectional KGSS conducted in 2014 because some variables were available only in that year [61].

### 4.1. Dependent Variables

There were two dependent variables, the first being perceived support for lowering the standard of living. The survey asked respondents, “How willing would you be to accept cuts in your standard of living in order to protect the environment?” The variable consisted of ordinal values ranging from 1 to 5. The other dependent variable was support for government spending for environmental protection. The respondents were asked whether they would like to see “more or less government spending” on the environment. This variable also consisted of ordinal values ranging from 1 to 5. These two variables were based on subjective single items. Single-item measures may not be ideal due to the lack of psychometric properties provided by multi-item measures. They were also limited in that they were subjective and deal with respondents’ behavioral intentions. These reflect the limitations of resorting to a secondary dataset, but some have argued that single-item measures are highly correlated with multi-item measures [62,63]. Similarly, there is a high correlation between subjective measures and objective measures [13,64].

### 4.2. Explanatory Variables

#### 4.2.1. Perceived Environmental Threats

This measure explored respondents’ perceptions of environmental threats from climate change and pollution. The measure comprised six items: “air pollution caused by cars,” “air pollution caused by the industry,” “pesticides and chemicals used in farming,” “pollution of Korea’s rivers, lakes, and streams,” “a rise in the global temperature caused by climate change,” and “gene modification in certain crops.” The respondents were given five choices to rate the perceived threat, with 1 indicating “not dangerous at all” and 5 indicating “extremely dangerous.” The reliability (Cronbach’s α) of the measure was 0.984. The mean of the measure was 3.62, suggesting that respondents, on average, were somewhat sensitive to environmental threats. Individuals perceiving a higher level of environmental threats would be likely to display pro-environmental behaviors, such as supporting the lower standard of living and higher government spending necessary for environmental protection [26,27].

#### 4.2.2. Political Participation

Political participation measured respondents’ self-assessment of their political lives. This measure comprised four items, with respondents asked to assess their participation in demonstrations, political rallies, online forums, and product boycotts “over the previous 12 months or for the period” [61]. Cronbach’s α for the measure was 0.951, indicating high consistency among the four items. The measure ranged from 1 to 4, and its mean was 1.67, suggesting that respondents, on average, were less likely to participate in political activities. Individuals with a higher level of political participation are expected to display high political efficacy [29,30] and increased support for a lower standard of living and government spending on environmental protection. More importantly, political participation is expected to serve as the moderator and enhancer of the relationship between perceived environmental threats and a lower standard of living as well as between perceived environmental threats and government spending on environmental protection, because political participation strengthens individuals’ mechanisms for coping with perceived environmental threats [49,52,60]. Table 1 describes the variables’ descriptive statistics.

### 4.3. Controls

The two models accounted for controls that explained individuals’ preferences for lower living standards and increased government spending for environmental protection. First, civic mindedness was included in the models. Studies have suggested that citizens who uphold civic principles display stronger pro-environmental preferences because they recognize that those issues endanger the society they hold dear [65]. The measure was formed by eight items. Respondents were asked about their self-assessment regarding such actions as participating in elections, paying taxes, tolerating others, helping others, and monitoring governmental actions. The measure was highly reliable, with a Cronbach’s α of 0.991. Second, the models accounted for political trust. The impact of political trust has been extensively studied in political science. Political trust functions as a heuristic, a mental shortcut through which individuals evaluate and support governmental policies and actions [66]. Those with a greater level of political trust likely give a particular policy the benefit of the doubt [66]. Thus, high-trust individuals would be likely to lend their support to government spending for environmental protection. Few studies have indicated a link between political trust and individuals’ choice to lower their standard of living. However, it is possible that high-trust individuals are less likely to be isolated from important contemporary issues such as climate change [67,68]. Therefore, they would likely recognize a need to solve the issues and view austere living more positively. Respondents were asked to assess their confidence in four political institutions: the executive branch of the national government, the local government, the National Assembly of Korea, and the Blue House (the president’s office, which is separate from the executive branch). The measure showed high consistency among the items (Cronbach’s α = 0.809). Third, the models controlled for individuals’ perceptions of pollution in their neighborhoods. Individuals who witness environmental problems near their places of residence are more likely to take ameliorative action [18]. The measure consisted of three items, touching upon respondents’ perceptions of air, water, and noise pollution near their homes; the measure had high reliability (Cronbach’s α = 0.989). Finally, the models accounted for the degree of political interest among the respondents. Individuals well-versed in contemporary political issues are more inclined to take environmental problems seriously and support ameliorative action [69]. The measure was based on a single item: how interested would you say you personally are in politics.

The models also accounted for respondents’ demographic characteristics. Studies have demonstrated that women are likely to be more sensitive to environmental issues than men [70]. Education plays a vital role in how individuals perceive progressive issues such as environmental protection. Highly educated individuals are more likely to be cognizant of issues such as climate change and pollution and more likely to understand what must be done to ameliorate these issues [71]. Income may cut both ways in its impact on pro-environmental behaviors. Individuals with higher incomes may want to live in a cleaner environment [72] and see the need for increased spending for the environment. However, they may not support increased spending on environmental protection that implies higher taxation [73]. Furthermore, they may feel that a more austere life is not in harmony with their income. Although age effects have been inconsistent across studies, a meta-study revealed that older individuals tend to show more concern about the environment and feel closer associations with nature and natural resources [74].

### 4.4. Measurement Validity

Harman’s single-factor test was performed to see whether a single factor explained the majority of the covariance in the models. The results indicated that no single factor explained more than 16.89% of the covariance, suggesting that common method variance did not threaten the validity of the models. Moreover, confirmative factor analysis (CFA) was conducted to identify model fits. The models consisted of five factors: perception of environmental threats, political participation, civic mindedness, political trust, and perception of local pollution. The CFA results yielded the following fit indices: $\chi^{2}$(265) = 1315.434, RMSEA = 0.055, SRMR = 0.038, CFI = 0.912, and TLI = 0.900. These index values met the threshold values [75] and indicated that the five-factor model fit the data well.

## 5. Results

The empirical analyses for the two models relied on an ordered probit. For the two ordinal dependent variables, running a linear regression may have resulted in biased estimates [76]. Using a binary probit method for the ordinal measures was possible, but it would shrink vital information contained in the ordinal measures [77]. Thus, an ordered probit was employed for the empirical models. The models included a weight that accounted for population representation [61].

The results are shown in Table 2. Model 1 had two steps. In Step 1, a direct relationship was examined between the explanatory variables and controls and individuals’ support for a lower standard of living. Step 2 was focused on the joint effects of environmental threat perception and political participation on support for a lower standard of living. Model 2 also had two steps. In Step 1, the direct relationships between the explanatory variables and controls and individuals’ support for government spending on environmental protection were examined. Step 2 was focused on the moderating effect of political participation on the link between environmental threat perception and support for government spending for environmental protection.

The results of Model 1 support Hypothesis 1a. Perceptions of environmental threats were positively associated with individuals’ support for a lower standard of living. Individuals with an acute sense of environmental threat are more likely to appraise them highly and at a higher degree of threat vulnerability and severity [38,39], and they would thus be motivated by the threats to pursue a lower standard of living for the sake of protecting the environment. The results also supported Hypothesis 2a. Politically engaged individuals are more likely to possess strong political efficacy [29,30,50] and to see that environmental protection requires a personal effort. Consequently, they would be supportive of a lower standard of living in exchange for environmental protection. Step 2 of Model 1 showed the joint effects of environmental threat perception and political participation on support for lower living standards. The results supported Hypothesis 3a, as political participation moderated and amplified the positive relationship between perceived environmental threats and support for a lower standard of living. Politically engaged individuals are more likely to have a greater degree of coping mechanisms [49,52] with which they channel their sensitivity to perceived environmental threats into proactive action, even if such action demands substantial sacrifices on their part, such as having a lower standard of living.

Similarly, the results for Model 2 supported Hypothesis 1b. Individuals with a high degree of threat severity and vulnerability induced by perceived environmental threats were more likely to support pro-environmental policies such as government spending [26,27]. Political participation, however, was not significantly associated with support for government spending for environmental protection. Political participation influenced individuals’ attitudes toward a lower standard of living; however, it did not affect individuals’ attitudes toward government spending. Nevertheless, political participation once again powerfully influenced the positive relationship between perceived environmental threats and support for government spending. Individuals with active political lives are more likely to feel a greater degree of political efficacy, which enables them to cope better with perceived environmental threats by taking ameliorative action, such as supporting government spending for environmental protection [49,52].

In terms of the control variables, civic mindedness predicted positive relationships with both dependent variables. Individuals abiding by civic principles are likely to recognize the threats that climate change and pollution pose to humanity and take action to counter them [65]. Political trust was positively associated with support for a lower standard of living. Individuals who place high trust in political institutions are likely to be liberals rather than conservatives [78], and they would support pro-environmental measures that involve personal efforts to change their lifestyles. Political interest was positively associated only with support for a lower standard of living. Individuals who have a strong interest in politics may understand their country’s environmental challenges [69] and thus be more supportive a more austere lifestyle to protect the environment. The relationship between education and support for a lower standard of living was not statistically significant. However, the relationship between education and support for government spending on environmental protection was significant. Better educated individuals may see that environmental issues require considerable effort and resources from the government [69], and this may encourage them to support the necessary government spending.

Table 3 displays predicted probabilities of individuals’ strong support for a lower standard of living and government spending. The results show the difference when the minimum and maximum values are set for each variable. Heightened perceptions of environmental threats increased the level of strong support for lowering one’s standard of living by 7.15% and for government spending for environmental protection by 37.85%. These results indicate that perceived environmental threats lead individuals to strongly support government spending while advocating a lower standard of living. Political participation increased strong support for a lower standard of living by 6.48% and for government spending by 4.47%. Unlike perceived environmental threats, political participation is slightly more effective in influencing people to change their personal lifestyles rather than support government spending. More importantly, however, perceived environmental threats and political participation became a formidable force when they were combined in their influence on strong support for the two dependent variables. When combined, the two variables increased strong support for a lower standard of living by 35.67% and for government spending by 69.58%.

**Figure 2:**
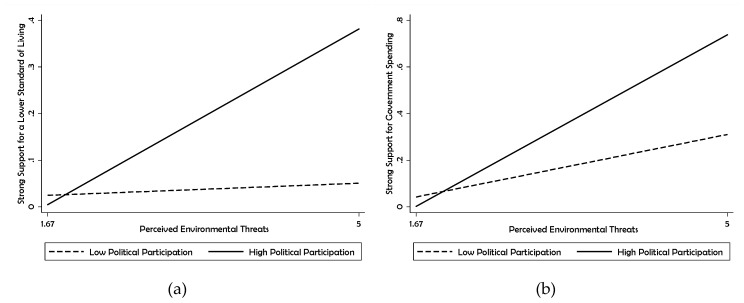
Predicted Probabilities of Strong Support for a Lower Standard of Living (**a**) and for Government Spending Necessary for Environmental Protection (**b**).

Figure 2 visualizes the moderation effect of political participation on the link between perceived environmental threats and strong support for a lower standard of living, as well as the link between perceived environmental threats and strong support for government spending on environmental protection.

The solid lines refer to the interactions between political participation and perceived environmental threats when levels of political participation are high (maximum); the dashed lines are designated for the interactions when political participation levels are low (minimum). In both cases, a higher degree of political participation substantially enhanced the influence of perceived environmental threats on the dependent variable, exhibiting steeper slopes. Additionally, the moderation effect of political participation was much stronger on the relationship between perceived environmental threats and “strong” support for government spending than on the relationship between perceived environmental threats and “strong” support for a lower standard of living.

## 6. Discussion

The results offer several intriguing points. First, as shown in Table 3, the direct effects of perceived environmental threats were strong. Perceived environmental threats increased strong support for government spending by more than 37%. The direct effects of political participation on strong support were weak. However, the study’s focus on the moderation of political participation is justified when looking at the statistically significant impact that political participation and perceived environmental threats jointly had on strong support of a lower standard of living (35.67%) and government spending (69.58%). This indicates that the way in which an individual perceives environmental threats is not a sufficient condition to maximize individuals’ pro-environmental behaviors. This study illustrated the possibility that political participation can function as a mechanism to turn an individual’s threat perceptions into tangible actions for pro-environmental outcomes, thus filling the gap between values and actions [31]. Even though individuals may have heightened threat perceptions, they may not act if they do not possess a stronger locus of control and sense of self-efficacy. Political participation can connect such missing links. Second, the moderation effects of political participation were stronger when individuals strongly supported government spending necessary for environmental protection than when they strongly supported a lower standard of living. This indicates that individuals’ political participation has more impact on individuals’ perceptions of government measures than on their support of a lower living standard. This has implications for policy makers who are concerned about pushing pro-environmental policies. Policy makers need to think about ways to reinvigorate citizens’ concerns about the environment and encourage citizens to express their concerns through various platforms, including open forums, demonstrations, and online forums. Politically active citizens have the strong sense of political efficacy necessary to reshape their lives and political outcomes. Having those citizens in the policy makers’ corner will likely aid the government’s efforts to pursue pro-environmental policies. Third, the study identified civic mindedness as an important factor in increasing individuals’ attitudes toward a lower standard of living and government spending. Individuals who uphold and tolerate laws may have strong proclivities toward ensuring the sustainability of the environment and human life [65], as their civic principles enable them to preserve their cherished community and environment. The substantial evidence in this study that civic-minded individuals exhibit pro-environmental behavior offers the possibility that civic mindedness serves not only as a direct influencer but also as a moderator and an amplifier of the link between environmental perceptions and pro-environmental behaviors.

The results have crucial implications for policy makers. First, public officials concerned about environmental issues must identify what makes citizens become sensitive to environmental threats. Public officials must make concerted efforts to ensure that citizens are informed about the state of environmental pollution, climate change, and other issues that impact the sustainability of human life. Public advertisement campaigns and mailed educational materials are adequate vehicles, but systemic educational attempts can be directed at young children. Much evidence shows that people who receive environmental education show pro-environmental preferences [18]. The results also show that education—although it was not environmental education—was a positive predictor of individuals’ support for government spending on environmental protection. Properly informed citizens would be much more perceptive to environmental threats, and this would enable them to lead environmentally friendly lifestyles and support government spending directed at environmental protection.

Second, public officials must consider carefully how to facilitate citizens’ participation in the public sphere and ensure that citizens develop a stronger sense of self-efficacy. Public officials may not welcome protests and rallies, but allowing citizens to express their voices in such settings will foster their political efficacy and increase their confidence to take action to ameliorate grave environmental threats. This may lead to an increased degree of personal fortitude in terms of reshaping their lifestyles and supporting governmental measures promising better environmental outcomes. Environmental issues are no longer fringe issues that can be ignored in favor of economic or social matters. The temperature is undeniably becoming warmer, and the frequency of environmental disasters is increasing [2,3]. Waiting for the government to respond to these problems will not solve the environmental challenges facing many countries. Citizens must be actively engaged in environmental discourse and taking actions that enhance the sustainability of human and natural life, and governments and citizens must work together to tackle these daunting challenges. Fostering citizens’ participation channels is one way to create a virtuous circle in which citizen support enables pro-environmental government policies, and the pro-environmental policies can be used in turn to facilitate exchanges among citizens with respect to environmental issues. Such actions may not solve the fundamental environmental challenges humankind is facing, but they can spark broad collaborations among domestic and international stakeholders. Additionally, environmentally minded public officials should be motivated to foster citizens’ self-efficacy, and in this education is once again vital. When individuals are exposed to opportunities for self-efficacy such as open-ended discussions, membership in politically conscious groups, and engagement in democratic decision-making processes, they develop the levels of political participation needed to facilitate environmental causes [19].

## 7. Conclusions

Climate change and environmental pollution are increasingly rendering the Earth uninhabitable [2,3]. Some contend that it is already too late to reverse the devastation wrought by industrial development and insatiable consumption, phenomena that commenced during the Industrial Revolution and accelerated over the last three decades [2,3]. Some countries will likely become uninhabitable due to rising temperatures, but humankind can at least mitigate the grim consequences of climate change and environmental pollution by practicing pro-environmental behaviors on the individual level.

In these increasingly perilous circumstances, this study investigated perceived environmental threats and political participation and examined how these factors individually and jointly influence support for a lower standard of living and government spending for environmental protection.

Finally, this study has some limitations. First, the study relied on a cross-sectional dataset surveyed in 2014. Moreover, the data for each individual were collected at the same time. Consequently, it is difficult to establish rigorous causality between explanatory and outcome variables. Second, the study used a single-source dataset and may not be completely free of the threats posed by common method variances. Future studies relying on datasets collected during different periods would alleviate some of the limitations inherent in a single-source cross-sectional dataset. Third, due to the use of secondary data, the study did not examine or observe actual behaviors, but relied on behavioral intentions. Although some argue that there are significant correlations between behavioral intentions and actual behaviors [13], more studies are needed to verify the mutuality in these relationships. Fourth, the models in this study investigated the more or less general outcomes of support for a lower standard of living and government spending. Determining whether the models would be applicable to more specific behaviors, such as reducing the use of plastic bags or driving less, will require further empirical verifications. Finally, we recognize that the political participation measure used in this study is not an ideal predictor for tapping into individuals’ political efficacy. Because the items related to political self-efficacy were not available from the survey data, we relied on political participation as an indirect measure. It is an imperfect measure whose impact as the moderator as in this study needs further verification in future studies as well.

## Figures and Tables

**Table 1:** Descriptive Statistics.

Variables	N	Mean	S.D.	Min.	Max.
Lower standard of living	758	3.03	1.14	1.00	5.00
Government spending necessary for environmental protection	759	3.81	0.81	1.00	5.00
Perceived environmental threats	759	3.62	0.63	1.67	5.00
Political participation	759	1.67	0.66	1.00	4.00
Civic mindedness	759	5.25	1.01	1.25	7.00
Political interest	759	2.44	0.78	1.00	4.00
Political trust	759	1.45	0.46	1.00	3.00
Perceived local pollution	759	2.45	0.63	1.00	4.00
Age	759	44.97	13.20	18.00	83.00
Female	759	0.42	0.49	0.00	1.00
Income	759	6.47	4.24	0.00	21.00
Education	759	4.00	1.40	0.00	7.00

**Table 2:** Regression Results.

	Support for a Lower Standard of Living				Support for Government Spending			
	Model 1 Step 1		Model 1 Step 2		Model 2 Step 1		Model 2 Step 2	
	Coef.	(S.E.)	Coef.	(S.E.)	Coef.	(S.E.)	Coef.	(S.E.)
Perceived environmental threats	0.21	0.08 ***	−0.10	0.19	0.50	0.08 ***	0.14	0.19
Political participation	0.19	0.07 ***	−0.55	0.36	0.08	0.07	−0.77	0.39
Perceived environmental threats × Political participation			0.20	0.10 **			0.23	0.11 **
Civic mindedness	0.22	0.05 ***	0.23	0.05 ***	0.14	0.04 ***	0.14	0.04 ***
Political interest	0.13	0.06 **	0.12	0.06 **	−0.09	0.06	−0.10	0.06
Political trust	0.18	0.10 *	0.17	0.10 *	−0.02	0.11	−0.02	0.11
Perceived local pollution	0.11	0.07	0.11	0.07	0.06	0.07	0.06	0.07
Age	0.00	0.00	0.00	0.00	0.00	0.00	0.00	0.00
Female	−0.02	0.09	−0.03	0.09	−0.13	0.09	−0.14	0.09
Income	0.01	0.01	0.01	0.01	0.00	0.01	0.00	0.01
Education	0.03	0.04	0.04	0.04	0.07	0.04 *	0.08	0.04 **
τ_1_	1.97	0.46	0.82	0.75	0.30	0.52	−1.04	0.79
τ_2_	3.04	0.47	1.89	0.76	1.24	0.47	−0.09	0.76
τ_3_	3.59	0.47	2.44	0.76	2.55	0.47	1.22	0.77
τ_4_	5.06	0.49	3.92	0.76	4.00	0.49	2.69	0.77
Log likelihood	−1052.93		−1050.56		−853.85		−850.97	
Wald test	83.91		86.09		65.44		70.00	
Number of cases	758				759			

* *p* < 0.1, ** *p* < 0.05, *** *p* < 0.01.

**Table 3:** Predicted Probabilities of Strong Support for a Lower Standard of Living and for Government Spending to Protect the Environment.

Variables	Strong Support for Green Living			Strong Support for Government Spending		
	Minimum	Maximum	Difference	Minimum	Maximum	Difference
Perceived environmental threats × political participation	2.46%	38.13%	35.67%	4.21%	73.79%	69.58%
Perceived environmental threats	1.75%	8.90%	7.15%	2.37%	40.22%	37.85%
Political participation	3.78%	10.26%	6.48%	15.65%	20.12%	4.47%
